# Diet‐induced DNA methylation within the hypothalamic arcuate nucleus and dysregulated leptin and insulin signaling in the pathophysiology of obesity

**DOI:** 10.1002/fsn3.1169

**Published:** 2019-09-05

**Authors:** Ebrahim Samodien, Carmen Pheiffer, Melisse Erasmus, Lawrence Mabasa, Johan Louw, Rabia Johnson

**Affiliations:** ^1^ Biomedical Research and Innovation Platform South African Medical Research Council. Tygerberg Cape Town South Africa; ^2^ Department of Medical Physiology Stellenbosch University Tygerberg South Africa; ^3^ Department of Biochemistry and Microbiology University of Zululand KwaDlangezwa South Africa

**Keywords:** arcuate nucleus, Diet‐induced obesity, DNA methylation, energy homeostasis, proopiomelanocortin

## Abstract

Obesity rates continue to rise in an unprecedented manner in what could be the most rapid population‐scale shift in human phenotype ever to occur. Increased consumption of unhealthy, calorie‐dense foods, coupled with sedentary lifestyles, is the main factor contributing to a positive energy balance and the development of obesity. Leptin and insulin are key hormones implicated in pathogenesis of this disorder and are crucial for controlling whole‐body energy homeostasis. Their respective function is mediated by the counterbalance of anorexigenic and orexigenic neurons located within the hypothalamic arcuate nucleus. Dysregulation of leptin and insulin signaling pathways within this brain region may contribute not only to the development of obesity, but also systemically affect the peripheral organs, thereby manifesting as metabolic diseases. Although the exact mechanisms detailing how these hypothalamic nuclei contribute to disease pathology are still unclear, increasing evidence suggests that altered DNA methylation may be involved. This review evaluates animal studies that have demonstrated diet‐induced DNA methylation changes in genes that regulate energy homeostasis within the arcuate nucleus, and elucidates possible mechanisms causing hypothalamic leptin and insulin resistance leading to the development of obesity and metabolic diseases.

## 1 INTRODUCTION

The global prevalence of obesity is rapidly increasing with an estimated 2.1 billion people considered overweight or obese (Tremmel, Gerdtham, Nilsson, & Saha, 2017). Obesity is a metabolic disorder that involves complex interactions between genetics and the environment and increases the risk for developing type 2 diabetes mellitus (T2DM), cardiovascular diseases, respiratory disorders, infertility, cancer, and social and psychological problems (Pozza & Isidori, 2018). The global economic cost attributed to obesity is approximated to be two trillion US dollars per annum, posing a significant health and financial burden, particularly to low‐ and middle‐income countries with already struggling healthcare systems (Tremmel et al., 2017).

The increased consumption of unhealthy, energy‐dense foods together with physical inactivity is widely regarded as the driving factors behind the current obesity epidemic. These factors are associated with energy imbalance due to prolonged energy intake exceeding energy expenditure, a hallmark of obesity (Romieu et al., 2017). Energy homeostasis involves co‐ordination of various metabolic signals emanating from the peripheral organs as well as the central nervous system (Lenard & Berthoud, 2008; Pimentel et al., 2012). Centrally, the hypothalamus plays an integral role in regulating whole‐body energy metabolism and controls many important physiological functions, with neurons of the arcuate nucleus (ARC) being essential for controlling food intake and energy expenditure (Lenard & Berthoud, 2008). The function of these neurons is modulated through the action of several hormones including leptin and insulin, both of which are involved in controlling energy homeostasis, playing crucial roles in regulating lipid and glucose metabolism, respectively (Belgardt & Bruning, 2010). Hypothalamic repression of their hormonal function results in dysregulated signaling, systemically affecting the major organs, manifesting as obesity, and metabolic syndrome (MeS) (Marino, Xu, & Hill, 2011; Morton & Schwartz, 2011).

Increasing evidence shows the involvement of diet‐induced epigenetic modulation of gene expression in the pathophysiology of obesity (Alegría‐Torres, Baccarelli, & Bollati, 2011). Indeed, overnutrition with unhealthy energy‐dense diets can elicit epigenetic alterations and thereby modulate gene expression (Vickers, 2014). Epigenetics can be defined as the study of changes in gene expression due to chromosome changes without any alterations occurring within the DNA sequence (Berger, Kouzarides, Shiekhattar, & Shilatifard, 2009; He et al., 2019). Epigenetic modifications include DNA methylation, noncoding RNAs, histone modification, and chromatin remodeling, all of which plays a significant role in regulating tissue‐specific gene expression (Holliday, 2006). Any modifications altering these processes can induce long‐term changes in gene function and metabolism, which may persist throughout the life of an individual and may be potentially inherited across generations (Sales, Ferguson‐Smith, & Patti, 2017).

A further understanding of leptin and insulin signaling within the hypothalamic ARC and the contribution of epigenetic mechanisms to the onset of obesity and related complications could aid in the development of novel antiobesity strategies. The mechanisms for maintaining energy homeostasis is complex, and several important hormones and neuropeptides are involved. This review will focus on DNA methylation as a possible mechanism regulating, specifically, leptin and insulin signaling within the hypothalamic ARC during the pathogenesis of obesity.

## 2 LEPTIN AND INSULIN AS REGULATORS OF ENERGY HOMEOSTASIS

**Figure 1:**
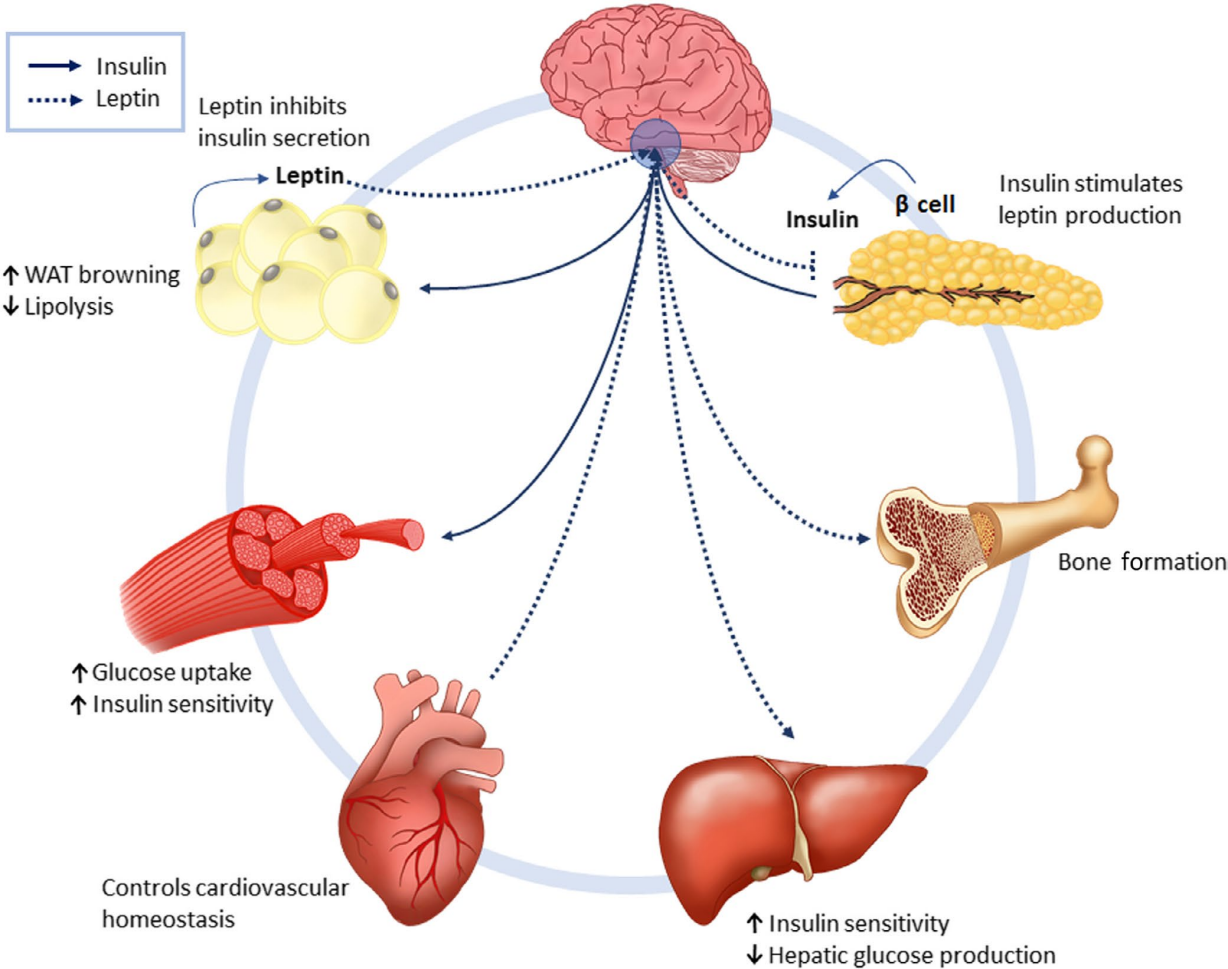
Leptin and insulin in whole‐body energy metabolism. Leptin and insulin have receptors within the hypothalamus to facilitate their role in regulating food intake. These hormones also have peripheral functions, such as controlling lipid and glucose metabolism within the liver, muscle, and adipose tissues. In the heart, leptin and insulin are involved in maintaining cardiovascular homeostasis and promoting cardiac efficiency. Leptin also plays a role in bone formation

Leptin and insulin are produced by the adipose tissues and pancreatic β cells in relation to lipid and blood glucose levels (Belgardt & Bruning, 2010). These are transported across the blood–brain barrier where they bind to the leptin (Lep‐R) and insulin receptors (Ins‐R), respectively, which are abundantly expressed within the ARC (Roh, Song, & Kim, 2016; Schwartz, Woods, Porte, Seeley, & Baskin, 2000). Apart from a common functional role in hypothalamic control of food intake and energy expenditure, leptin signaling and insulin signaling are involved in controlling important functions within the major organs as depicted in Figure 1.

In the peripheral tissues, insulin is the primary signal for fat storage (Isganaitis & Lustig, 2005) and is involved in regulating blood glucose levels through increasing whole‐body glucose uptake (Roder, Wu, Liu, & Han, 2016). Leptin, in turn, regulates hepatic gluconeogenesis, skeletal muscle glucose uptake, and lipid oxidation (Paz‐Filho et al., 2012). Insulin inhibits lipolysis and promotes thermogenesis in brown adipose tissue (Sanchez‐Alavez et al., 2010), while leptin promotes glucose uptake in brown adipose tissue and tissue lipolysis in white adipose tissue (Park, Ahn, & Kim, 2010; Sanchez‐Lasheras, Konner, & Bruning, 2010). In the heart, insulin affects substrate utilization through promoting glucose metabolism through glycolysis, which diminishes myocardial O_2_ consumption and promotes cardiac efficiency (El‐Shenawy, Moharram, El‐Noamany, & El‐Gohary, 2013). Under pathological conditions such as T2DM, myocardial ischemia, and cardiac hypertrophy, insulin signal transduction pathways are abrogated (Iliadis, Kadoglou, & Didangelos, 2011).

Leptin also functions to inhibit inflammation and enhance the immune response, plays a role in coagulation, neurogenesis, platelet aggregation, fibrinolysis, affects reproduction and controls cardiovascular homeostasis, and is involved in tissue remodeling as well as bone formation (Chehab, 2014; Kelesidis, Kelesidis, Chou, & Mantzoros, 2010; Koh, Park, & Quon, 2008; McNay, Briancon, Kokoeva, Maratos‐Flier, & Flier, 2012; Paz‐Filho, Mastronardi, Delibasi, Wong, & Licinio, 2010; Paz‐Filho, Wong, & Licinio, 2011; Upadhyay, Farr, & Mantzoros, 2015). Perturbed leptin signaling stimulates oxidative stress, vascular inflammation, and vascular smooth muscle hypertrophy, that may contribute to the pathogenesis of T2DM, hypertension, atherosclerosis, and coronary heart disease (Bell, Harlan, Morgan, Guo, & Rahmouni, 2018; Koh et al., 2008).

It could be hypothesized that functional resistance to leptin and insulin within the hypothalamic ARC results in leptinemia and insulinemia, thereby affecting the peripheral tissues subsequently manifesting as obesity and related metabolic diseases.

## 3 THE ARCUATE NUCLEUS AND ENERGY HOMEOSTASIS

**Figure 2:**
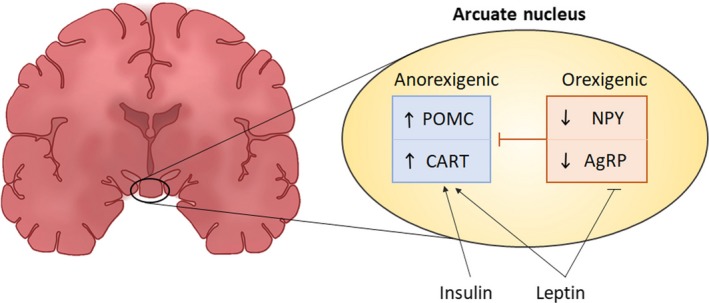
Hypothalamic arcuate nucleus and energy homeostasis. During a normal physiological state, levels of insulin and leptin rise after a meal and elicit the anorexigenic neurons to release CART and POMC triggering satiety. Leptin also suppresses the orexigenic NPY and AgRP neurons to decrease appetite

The ARC is located within the mediobasal hypothalamus and is the critical brain region regulating energy homeostasis (McNay et al., 2012). It is optimally positioned for its function and is often described as a “window” to sense nutrients and hormonal signals in circulation and therefore able to mediate appropriate metabolic responses (Langlet, 2014; Rahmouni, 2016). The ARC contains both anorexigenic and orexigenic neurons which perform opposing functions in order to control appetite and regulate energy metabolism (Benite‐Ribeiro, Putt, Soares‐Filho, & Santos, 2016). The anorexigenic neurons act to promote satiety, while the orexigenic neurons stimulate appetite, which is achieved through combined functions of insulin and leptin and their stimulation of these respective neurons. The neural–hormonal circuitry between leptin and insulin and the ARC neurons controlling food intake and energy metabolism is illustrated in Figure 2.

These neuronal populations are characterized by the expression of specific neuropeptides that have potent effects on energy homeostasis (Bell et al., 2018; Varela & Horvath, 2012). The anorexigenic effect is elicited through the action of proopiomelanocortin (POMC) and cocaine‐ and amphetamine‐regulated transcript (CART), which are neuropeptides known to decrease food intake and body weight (Millington, 2007). Opposingly, agouti‐related peptide (AgRP) and neuropeptide Y (NPY) mediate an orexigenic effect, which stimulates appetite (Varela & Horvath, 2012).

The mechanisms detailing leptin and insulin signaling within the hypothalamic ARC have been reviewed by Varela and Horvath (2012). Briefly, after a meal, the rise in blood glucose levels causes an increase in the secretion of insulin from pancreatic β cells, which then stimulate leptin synthesis and secretion from adipose tissues (Paz‐Filho et al., 2012). The increase in leptin and insulin levels elicits the anorexigenic neurons to express CART and POMC, which then produces α‐melanocyte‐stimulating hormone, thereby triggering satiety and increasing energy expenditure (Millington, 2007). Simultaneously, leptin suppresses the orexigenic neurons NPY and AgRP, thereby decreasing appetite (Coll, Farooqi, & O'Rahilly, 2007). In a fasted state, there is a reduction in insulin and leptin levels which causes the orexigenic neurons to secrete NPY and AgRP, thereby stimulating appetite and increasing energy intake (Timper & Brüning, 2017). During obesity, there is an imbalance between insulin‐stimulated leptin‐induced anorexigenic and orexigenic signaling. As such, an overexpression of NPY/AgRP with a concomitant reduction of POMC/CART results in a diminished satiety signal that leads to an increased appetite and decreased energy expenditure (Bergen, Mizuno, Taylor, & Mobbs, 1999).

## 4 DNA METHYLATION IN OBESITY

Obesity can be defined as a disorder in which excess body fat accumulation is accompanied by profound changes in physiological function (Kopelman, 2000). It represents a modifiable risk factor that is an underlying and reoccurring theme within development of several major noncommunicable diseases. The early onset of obesity drastically increases the likelihood of developing related disorders later in life (Dietz, 1994).

In recent times, the epigenetic control of gene expression has been increasingly investigated as a mechanism to explain the etiology of this complex disease (He et al., 2019). Accumulating evidences has implicated altered DNA methylation profiles with the development of various human diseases, including obesity as well as T2DM, cardiovascular disease, cancer, and neurodegenerative diseases (Davegårdh, García‐Calzón, Bacos, & Ling, 2018; Koch et al., 2018; Lopomo, Burgio, & Migliore, 2016; Lu, Liu, Deng, & Qing, 2013; Portela & Esteller, 2010; Zhang & Zeng, 2016). DNA methylation is the most extensively studied and best characterized epigenetic mechanism and involves the addition of a methyl group to the 5th carbon of the cytosine pyrimidine ring within CpG nucleotides by DNA methyltransferases (DNMTs) (Krasilnikova et al., 2018; Weinhold, 2006). DNA methylation often occurs within regulatory regions of genes and serves as an impasse for transcription factors and the transcriptional machinery, thereby modulating gene expression (Long, Smiraglia, & Campbell, 2017). The epigenetic phenotype can be influenced by environmental factors, such as nutrition, physical activity, working habits, behavior, stress, smoking, and alcohol consumption (Alegría‐Torres et al., 2011).

It has long been hypothesized that adverse nutritional environment *in utero* can cause deficiencies in body organs development, resulting in “programmed” susceptibility that interacts with later diet and environmental stressors to cause overt disease many decades later (Barker, Osmond, Golding, Kuh, & Wadsworth, 1989). Maternal diet directly influences offspring epigenome (Keleher et al., 2018; Oestreich & Moley, 2017; Wolff, Kodell, Moore, & Cooney, 1998) and thus the genetic background, which can confer susceptibility to disease development. It is estimated that 40%–70% of the variation in obesity‐related phenotypes in humans is heritable (Bell, Walley, & Froguel, 2005; He et al., 2019; Herrera, Keildson, & Lindgren, 2011).

Epigenetic inheritance can be defined as the transmission of certain epigenetic marks to offspring (van Otterdijk & Michels, 2016), which can be classified by the mode of transmission and whether it elicits a direct or indirect effect, that is, resulting due to factors occurring during the lifetime of the individual or whether it occurs due to events experienced by the parents or grandparents (Lacal & Ventura, 2018). How these epigenetic transfers occur intra‐ (during a generation) and transgenerationally (across generations) is intriguing, with the latter occurring through separate pathways, such as intrauterine conditions or through gametes (Lacal & Ventura, 2018). The mechanism of transmission is not limited to the transfer of DNA, but can also occur through noncoding RNAs, as well as certain protein and metabolites (Perez & Lehner, 2019). There is an increasing body of research available, related to the role of sperm‐ and ejaculate‐mediated mechanisms of transgenerational epigenetic transfer, illustrating the importance of paternal contribution in this regard (Crean & Bonduriansky, 2014; Jablonka & Raz, 2009). Overwhelmingly, most research has evaluated maternal role in epigenetic inheritance (Soubry, 2015), especially with regard to DNA methylation and its role in obesity pathophysiology.

Such studies have revealed that offspring of obese mothers exhibit elevated body fat, leptin cord blood, and cytokine levels (Catalano, Presley, Minium, & Hauguel‐de Mouzon, 2009), together with increased insulin, cholesterol, and blood pressure (Gaillard, 2015). The effects of maternal obesity persist into adulthood, increasing the risk for cancer, metabolic disorders (Glastras, Chen, Pollock, & Saad, 2018), impaired cognitive and executive functions, and neuropsychiatric problems (Mina et al., 2017; Pugh et al., 2015). The effects of high‐fat diet (HFD) and obesity in mice are similar to those in humans (Keleher et al., 2018). Numerous animal studies have employed dietary manipulation in rodent models to evaluate the effects of maternal obesity on offspring, many of which have supported the concept that maternal obesity programs the development of metabolic disease in adulthood and compounds the effects of diet‐induced obesity (Glastras et al.., 2018). C57BL/6J and C57BL/CNG1 mice exposed to HFD during gestation have been shown to exhibit increased body weight, hyperglycemia, hypertension, fatty accumulation in the liver, and insulin resistance (IR) as adults, even when fed a standard diet after birth (Elahi et al., 2009; Liang, Oest, & Prater, 2009). The largest meta‐analysis of animal studies included 53 rodent studies confirming that maternal obesity was associated with higher body weight in offspring (Lagisz et al., 2015).

The effect of obesogenic diets on altered DNA methylation during the pathogenesis of obesity is well documented; however, studies evaluating the effect of such diets on epigenetic neuronal signaling are limited. Understanding the contribution of diet‐induced DNA methylation modifications occurring within the brain during the development of obesity could lead to risk‐stratification for targeted therapeutic intervention (Feinberg, 2007).

## 5 DIET‐INDUCED DNA METHYLATION WITHIN THE ARCUATE NUCLEUS

Challenging socioeconomic conditions increases the likelihood of being overweight or obese, being directly associated with the cost of healthy food and ultimately leading to less nutritious food choices (Pechey & Monsivais, 2016). Such adverse socioeconomic and even psychosocial factors can directly alter gene expression through epigenetic‐mediated pathways, leading to the development of obesity and related conditions such as excessive visceral fat accumulation, IR, dislipidemia, hypertension, and cardiocerebral diseases (Paternain et al., 2012; Rosmond & Bjorntorp, 1999). Several studies using rodents were conducted with the aim of elucidating the effect of obesogenic diets on arcuate nucleus DNA methylation and gene expression profiles, with these being summarized in Table 1.

**Table 1:** Animal studies examining the effect of obesogenic diets on DNA methylation and gene expression profiles within the arcuate nucleus

	Study design	∆ DNA methylation	∆ Gene expression	Biological response	References
Direct epigenetic transmission					
Wistar Rat Male	Investigated the effect of HFD consumption postweaning to adulthood (days 21 to 90) on POMC promoter DNA methylation	↑ *POMC*	No change POMC	↑ BW ↑ leptin ↑ insulin	Marco et al. (2013)
Sprague Dawley Rat Male	Investigate the sensitivity/resistance to weight gain through measuring hypothalamic neuropeptide transcription	↑ *POMC* No change *NPY*	↓ POMC No change NPY, AgRP, and PPAR‐γ	↑ BW, ↑ energy intake obese phenotype	Cifani et al. (2015)
Wistar Rat Female	Examine DNA methylation and mRNA levels of feeding‐related neuropeptides in female rats fed CD for 20 weeks	*NPY* unmethylated *POMC* unmethylated	↑ NPY within PVN and VMN ↑ POMC within ARC	↑ energy intake, ↑ BW ↑ fat depots but no MeS	Lazzarino et al. (2017)
Indirect epigenetic transmission					
Wistar Rat Female	Assess the hypothalamic response of high‐fat‐sucrose (HFS) diet in female rats exposed to prenatal stress (PNS)	↑ *SLC6A3* ↑ *POMC*	↓ SLC6A3, ↓ NPY, ↓ INS‐R ↑ POMC	PNS ↑ energy intake ↓ birth weight ↓ body length, no change in fat mass PNS worsened obesity ↑ adiposity ↑ insulin	Paternain et al. (2012)
Wistar Rat	Induce neonatal overfeeding through reduced litter size and mapping hypothalamic POMC promoter DNA methylation	↑ *POMC*	↓ POMC	↑ BW and MeS ↑ leptin ↑ insulin	Plagemann et al. (2009)
Kunming mice	Investigate the importance of Sp1‐binding site for leptin‐mediated POMC activation in cultured cells and postnatal mice reared by dams fed CLAs	↑ *POMC*	↓ POMC	*↑* energy intake ↑ glucose ↓ insulin sensitivity	Zhang et al. (2014)
Transgenic Mice	Investigate the role of MeCP2 in regulating POMC expression in POMC specific MeCP2‐KO mice, through characterizing POMC promoter methylation	↑ *POMC*	↓ POMC	↑ BW ↑ fat mass ↑ food intake ↑ respiratory exchange ratio	Wang et al., (2014)
C57BL/6J Male mice	Determine DNA methylation and gene expression of POMC and MC4R in offspring of dams fed high‐fat high‐sugar diet during pregnancy and lactation	No change *MC4R* ↓ *POMC*	↑ MC4R ↑ POMC	↑ BW impaired glucose tolerance ↓ insulin sensitivity and ↑ leptin	Zheng et al., (2015)
Sprague Dawley Rat	Investigate the long‐term effects of maternal HFD on hypothalamic *Pomc* methylation of offspring fed LFD or HFD for 20 weeks	↑ *POMC*	No change in POMC ↓ NPY and AgRP	↑ BW ↑ adiposity ↑ leptin	Gali Ramamoorthy et al. (2018)
Sprague Dawley Rat	Determine sex‐specific effects of HFD on before and during pregnancy and lactation on hypothalamic genes	↑ *INS‐R*	↓ INS‐R No change in LEP‐R and GLUT‐3	↑ BW ↑ leptin ↑ insulin impaired OGTTs in both sexes ↑ insulin resistance in males only	Schellong et al. (2019)
Wistar Rat	Investigated methylation of insulin receptor promoter in overfed neonatal pups	↑ *INS‐R*	↓ INS‐R	↑ BW and MeS ↑ leptin ↑ insulin	Plagemann et al. (2010)

Abbreviations: AgRP, agouti‐related protein; ARC, arcuate nucleus; CD, cafeteria diet; CLAs, conjugated linoleic acids; GLUT‐3, glucose transporter 3; HFD, high‐fat diet; HFS, high‐fat‐sucrose; INS‐R, insulin receptor; KO, knockout; LEP‐R, leptin receptor; LFD, low‐fat diet; MC4R, melanocortin 4 receptor; MeCP2, methyl‐CpG binding protein 2; MeS, metabolic syndrome; NPY, neuropeptide Y; PNS, prenatal stress; POMC, proopiomelanocortin; PPAR‐γ, Peroxisome proliferator‐activated receptor gamma; PVN, paraventricular nucleus; SLC6A3, dopamine active transporter; VMN, ventromedial nucleus.

In a study where male rats were fed HFD postweaning into adulthood, these animals exhibited increased BW with elevated plasma leptin and insulin levels, in addition to increased *POMC* methylation without any changes in POMC mRNA expression levels (Marco, Kisliouk, Weller, & Meiri, 2013). In another report where the susceptibility or resistance to diet‐induced obesity (DIO) was investigated, five‐week‐old male Sprague Dawley rats were fed HFD with changes hypothalamic gene expression measured. After 21 weeks of HFD‐feeding, no changes in NPY or AgRP expression level between the respective groups were observed, while a reduced expression of POMC within the DIO group was reported when compared to the diet resistant group. Furthermore, no changes in *NPY* methylation were observed, while *POMC* promoter methylation was reduced in diet resistant group compared with the DIO group (Cifani et al., 2015). These studies provide evidence for obesogenic diet‐induced alteration in DNA methylation of arcuate nucleus genes, such as *POMC*, which were associated with raised leptin and insulin levels and the development of obesity and MeS (Cifani et al., 2015; Marco et al., 2013).

There has been a gender bias in the literature, with mostly males being used in experimental settings, particular within neurological sciences and a call has been made for this to be addressed (Coiro & Pollak, 2019). Several reports of female rats being resistant to DIO, which has been attributed to sex hormones (Giles, Jackman, & MacLean, 2016). With the fluctuations of the estrous cycle adding to the complexity, investigating such phenomenon is challenging. Nevertheless, it is important to perform experiments including females, which will aid in the greater understanding of gender disparities in disease and in the quest for developing effective therapeutics.

In a study by Lazzarino et al. (2017), female rats were fed a cafeteria diet from weaning for 20 weeks. These animals displayed an increase in BW and presented with increased fat depots but interestingly, they did not develop MeS (Lazzarino et al., 2017). Methylation analysis revealed that the *NPY* promoter was unmethylated at an activating enhancer binding protein‐2 alpha TF‐binding site, resulting in an increase in energy intake albeit due to an orexigenic signal emanating from neighboring nuclei within the ventromedial nucleus and paraventricular nucleus. However, in the ARC, the *POMC* promoter was unmethylated in a *Mae II* site adjacent to a signal transducer and activator of transcription 3 (STAT‐3) TF‐binding site. POMC is activated by STAT‐3 in response to leptin signaling through specificity protein‐1 (Sp1) promoter binding, and decreased *POMC* promoter methylation enables TF‐binding to facilitate leptin action within the ARC neurons (Lazzarino et al., 2017). The observed increase in POMC coupled with reduced AgRP expression was suggested to counteract the orexigenic signal from the neighboring nuclei thus limiting weight gain. Although MeS was not observed, these animals did exhibit increased adiposity and BW, as well as altered *POMC* methylation. The reported compensatory mechanism to limit weight gain in the study of Lazzarino et al. (2017) is intriguing and necessitates the need for similar female studies to further investigate such scenarios.

## 6 INHERITABLE ARCUATE NUCLEUS EPIGENETIC PROGRAMMING

Epigenetic marks can be inherited across a generation (Lacal & Ventura, 2018); thus, to investigate whether an obesogenic diet induces intergenerational effects on arcuate nucleus DNA methylation, several studies exposing offspring to a challenging *in utero* environment have been conducted. In a study evaluating the effect of prenatal stress on the susceptibility of offspring to high‐fat‐sucrose (HFS) diet‐induced obesity in adult female offspring, it was found that stress alone induced lower birth weight and body length without affecting body fat mass, while also resulting in an increased energy intake in adulthood (Paternain et al., 2012). HFS‐diet feeding induced obesity, increased adiposity, and IR, which was worsened in the stress‐induced group as compared to the nonstressed DIO group. DNA methylation analysis revealed that the dopamine active transporter (*SLC6A3*) gene promoter was hypermethylated at CpG site −37 bp from the transcriptional start site. This gene is related to the central reward neurocircuitry system and is indicative of a mechanism whereby early‐life stressors contribute to hedonistic cues that influence feeding behavior that may override metabolic demands (Paternain et al., 2012; Vucetic, Carlin, Totoki, & Reyes, 2012), with it being well known that a heightened dopamine action within the hypothalamus promotes food intake (Meguid et al., 2000). Furthermore, *POMC* was found to be hypermethylated at CpG site −167 bp, but only within the nonstressed animals. This could perhaps be due to the reproductive hormones potentially conferring a protective response (Puerta, Nava, Abelenda, & Fernandez, 1990). Estradiol action is achieved through binding to estrogen receptor, which is abundantly expressed within POMC neurons in the ARC and provides a means to potentiate leptin and or insulin signaling (Frank, Brown, & Clegg, 2014; Lazzarino et al., 2017; Mauvais‐Jarvis, Clegg, & Hevener, 2013; de Souza et al., 2011; Torre, Benedusi, Fontana, & Maggi, 2013). With the induction of stress, the estradiol‐mediated response could have been upregulated, leading to no *POMC* methylation alterations being observed within the stressed group. Although this was not investigated in this study, evaluating the role of estradiol in facilitating this effect, for the purpose of future modification, could be valuable.

Several other animal studies have implicated altered *POMC* methylation in obesity‐related metabolic disease development. Indeed, overfed Wistar rat offspring exhibited an increase in *POMC* promoter hypermethylation at two specific sites, which were shown to be essential for leptin and insulin action (Plagemann et al., 2009). In a study analyzing mice offspring of dams fed conjugated linoleic acids (CLAs) to induce metabolic disorders, the CpG sites −100 and −103 bp of the Sp1‐binding site were found to be crucial for POMC expression (Zhang et al., 2014). Sp1 is a ubiquitous zinc‐finger protein that binds GC‐rich elements in the promoters of many housekeeping as well as tissue‐specific genes (Paonessa, Latifi, Scarongella, Cesca, & Benfenati, 2013; Philipsen & Suske, 1999). Using an in vitro approach, it was further demonstrated that methylation blocked the formation of the Sp1‐promoter complex, thereby inhibiting leptin‐mediated POMC activation (Zhang et al., 2014).

Similarly, nuclear methyl‐CpG binding protein 2 **(**MeCP2) has also been demonstrated to be important for POMC expression. Using mice with MeCP2 deletion in hypothalamic POMC neurons, Wang and colleagues showed that *POMC* promoter methylation was increased leading to reduced POMC expression, with these animals being overweight and exhibited increased adiposity, food intake, and respiratory exchange ratio as well as elevated leptin levels. In this study, a functional synergism was observed between MeCP2 and cAMP responsive element binding protein 1 in positively regulating the *POMC* promoter (Wang, Lacza, Sun, & Han, 2014). Furthermore, in a study by Zheng et al. (2015), offspring of dams fed a high‐fat high‐sugar diet during pregnancy, lactation, and postweaning (for a period of 32 weeks), exhibited an increase in BW, impaired glucose tolerance, decreased insulin sensitivity, and increased serum leptin, all of which correlated with increased POMC and MC4R expression (Zheng et al., 2015). However, no change in melanocortin 4 receptor (*MC4R*) methylation levels was detected, while consistent hypomethylation of *POMC* promoter was observed which was negatively associated with glucose response.

In a recent study investigating the long‐term impact of maternal HFD on offspring susceptibility to developing obesity, *POMC* methylation was investigated at weaning and after 20 weeks of exposure to either a low‐fat diet (LFD) or HFD (Gali Ramamoorthy et al., 2018). The offspring of dams fed HFD had increased BW and adiposity as well as elevated leptin levels at weaning. In the offspring subsequently fed LFD, these animals retained the increased BW, while animals fed HFD were severely hyperphagic and exhibited rapid weight gain with increased fat mass and IR. ARC gene expression analysis showed that NPY and AgRP were downregulated, while POMC expression was unchanged. Methylation analysis revealed *POMC* methylation at neural enhancer 1 and neural enhancer 2 (nPE1 and nPE2) region and at the Sp1 site within the *POMC* promoter, with *POMC*‐promoter‐Sp1 methylation being retained into adulthood. These finding highlights the long‐term effect of the maternal diet on the offspring's epigenetic phenotype related to impaired energy homeostasis, through disruption of POMC and leptin‐mediated signaling within the ARC.

In a study by Schellong and colleagues, the objective was to discern the contribution of sex differences to the susceptibility to DIO (Schellong et al., 2019). In this study, both males and female offspring from dams fed HFD before and during pregnancy and lactation were evaluated. The MeS phenotype was induced in both sexes; however, gender‐specific epigenetic alterations were observed. DNA methylation analysis revealed increased methylation at the insulin receptor nuclear factor CpG site of the *INS‐R gene* promoter, leading to decreased Ins‐R expression, consequently resulting in higher levels of IR within the male rats only (Schellong et al., 2019). These findings are supported by a previous study by Plagemann and colleagues, who also demonstrated hypermethylation of the hypothalamic *INS‐R* promoter methylation in response to obesogenic diet. These animals also presented with symptoms of MeS, including obesity, hyperleptinemia, hyperglycemia, and IR (Plagemann et al., 2010). These observations could offer an alternate but complementary mechanism to the *POMC*‐mediated methylation pathway, resulting in altered neuropeptide expression leading to the MeS‐like phenotype (Schellong et al., 2019).

Collectively, these studies demonstrate how obesogenic diet predisposes the offspring to early‐onset obesity, glucose insensitivity, and IR later in life. These effects are associated with alteration in epigenetic patterns that influence the expression of important genes within the ARC. The obesogenic diet‐induced effects seen within these studies are not always consistent, regarding the effect on methylation as well as the resultant gene expression changes. It was also not always possible to validate the mRNA expression with the protein levels, which could be important due to post‐translational modification. It is also feasible that these differing effects could be related to the age of the animals and the timepoint at which the diet was administered, as well as the length of dietary exposure, the type of fat used, and the epigenetic phenotypic background of the animals. This perhaps points to the fact that more standardization is required in terms of how the experimentation is performed. Sex differences in response to diet are also known to occur and could surely affect the outcome. Accumulating evidence related to the paternal transmission of epigenetic information is becoming available, having widespread effects, with this too certainly impacting the epigenetic phenotypic background and therefore requires that the role of paternal epigenetic transmission in this regard to be taken into consideration when conducting this type of research.

## 7 POMC METHYLATION AND OBESITY

**Figure 3:**
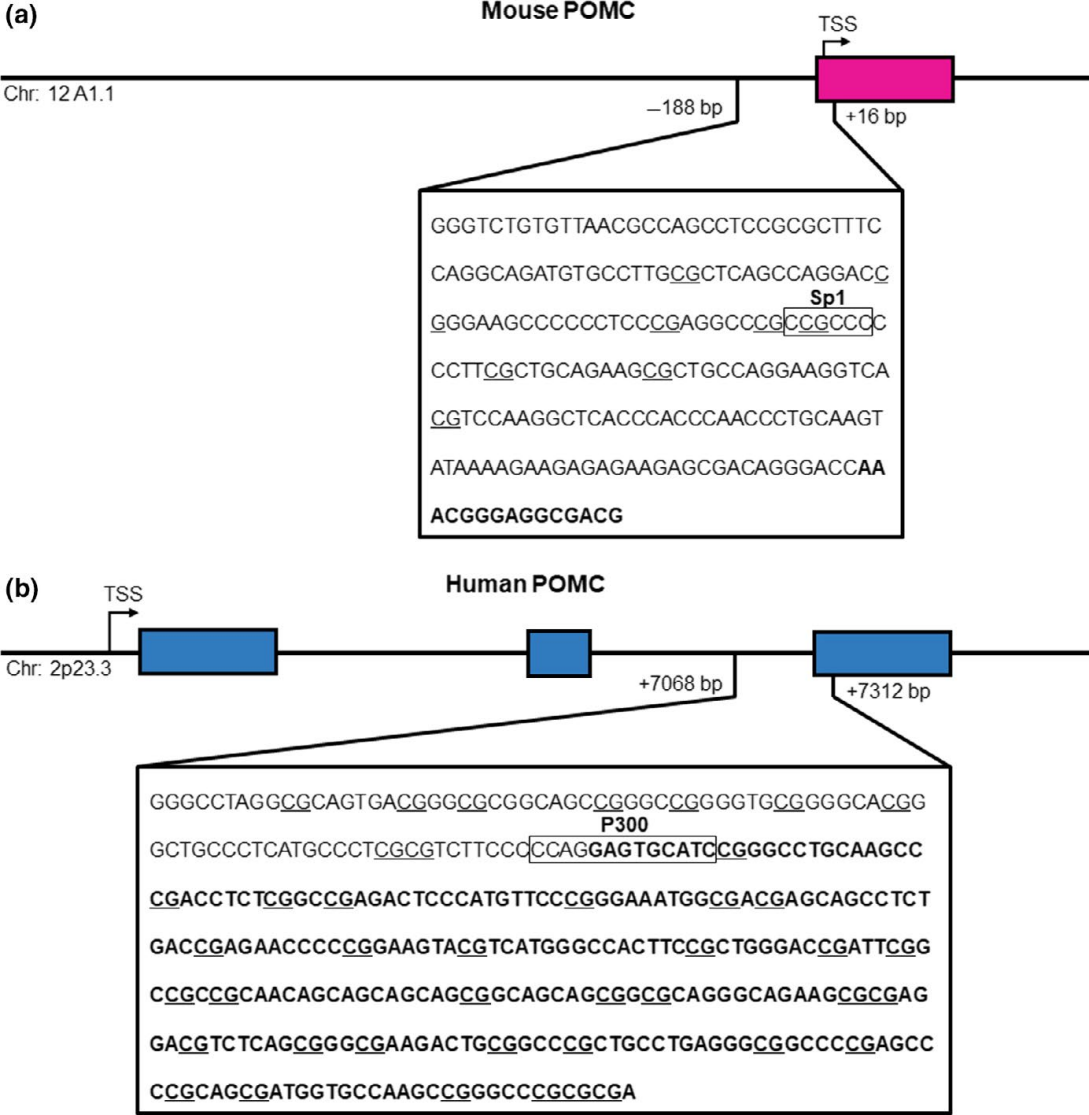
*POMC* methylation in obesity. The studies by Zhang et al., (2014) and Kuhnen et al. (2016), have shown that hypermethylation of transcription factor binding sites for Sp1 and P300 within *POMC* results in repressed gene expression. Sequences of the (a) mouse and (b) human *POMC* gene were obtained from Ensembl (GRCh38.p12 and GRCm38.p6 builds, respectively). The chromosome location, the transcriptional start site, intronic regions (pink or blue), exonic regions (bolded letters), and putative CpG sites (underlined) are indicated

The importance of *POMC* methylation status in obesity pathology cannot be overstated, with this locus being identified along with several other candidate genes in linking maternal nutrient exposure to adverse health in human intergenerational studies (James et al., 2018). All sequenced mammalian *POMC* genes consist of three exons, separated by two large introns (Millington, 2007). In humans, *POMC* methylation is established in the early embryonic stage, influences body mass index, and is regarded as the cause of, rather than a consequence of, obesity (Kuhnen et al., 2016). As in animals, the methylation status at specific CpG sites is important for TF‐binding and can thereby inhibit POMC transcription (Kuehnen et al., 2012; Zhang et al., 2014). In humans, methylation occurring at the intron 2/exon 3 border of *POMC* has also been implicated in reduced P300 TF‐binding and thereby *POMC* transduction (Kuehnen et al., 2012). The methylated regions of *POMC* within mice and human genes, associated with reduced TF‐binding, are illustrated in Figure 3.

*POMC* methylation, particularly on the Sp1 binding site within the promoter, is consistently linked with disease pathology in both intrauterine and postweaning exposure to obesogenic diets. *POMC* controls several important physiological processes such as lactation, regulation of sexual behavior, reproductive cycles, and sexual function as well as mediating the immune system and stress response and possibly central cardiovascular functioning (Catania, Airaghi, Colombo, & Lipton, 2000; Millington & Buckingham, 1992; Van der Ploeg et al., 2002).

In the studies evaluated herein, no methylation changes were observed for *NPY*, *MC4R,* or *PPAR‐*γ when investigated (Cifani et al., 2015; Zheng et al., 2015), while the methylation status of *POMC* was consistently found to be altered and positively associated with increased levels of leptin and insulin and the development of the obesity phenotype and symptoms of MeS (Cifani et al., 2015; Gali Ramamoorthy et al., 2018; Marco et al., 2013; Andreas Plagemann et al., 2009; Wang et al., 2014; Zhang et al., 2014; Zheng et al., 2015). With POMC‐mediated leptin and insulin function playing a major role in the pathological development of obesity and MeS, the methylation status of *POMC* thus represents a pivotal therapeutic target.

## 8 LIFESTYLE MODIFICATION INTERVENTIONS

As the obesity epidemic rages on, with many obesity drugs available but no real effective treatment, the pursuit for therapeutics continues. From a gene‐culture evolutionary perspective, the global increase in obesity prevalence is unprecedented, perhaps representing the most rapid population‐scale shift in human phenotype ever to occur (Bentley, Ormerod, & Ruck, 2018). Lifestyle modification to date remains the best way to achieve healthy outcomes, with diet and exercise being critically important, with such preventative approaches being astoundingly underrepresented (Schellong et al., 2019). Exercise is a powerful antiobesity tool, with several studies indicating that exercise can have positive outcomes through affecting epigenetics (Brown, 2015; Nitert et al., 2012; Ronn et al., 2013). In a study using mice, it was reported that exercise had positive effects on restoring POMC action impaired by HFD (Laing et al., 2016). It would be interesting to investigate whether this effect was achieved through modulation of *POMC* DNA methylation patterns.

He et al. (2018) recently demonstrated, utilizing neuron‐specific transgenic mice models for patch‐clamp electrophysiology experiments, that exercise depolarized and increased the firing rate of arcuate nucleus POMC neurons, with leptin playing an integral role in the synaptic reorganization (He et al., 2018). POMC neurons expressing leptin receptors were found to have increased sensitivity to exercise‐induced biophysical changes, while opposingly, NPY neurons were inhibited postexercise (He et al., 2018).

Diet is equally important, especially nutrient‐rich diets containing elevated levels of phytochemicals, which has been associated with reduced incidence of cardiovascular disease, cancer, neurodegenerative diseases, and psychiatric disorders among others (Gomez‐Pinilla & Nguyen, 2012). Phytochemicals are natural compounds that have been increasingly investigated for their health benefits and with further inquiry could prove these to be cheaper alternative therapies. Many of such nutraceuticals are widely available and have been proven to be safe, often eliciting beneficial effects in modulating disease by the activation of various protective mechanisms (Vasileva, Marchev, & Georgiev, 2018)**.**

Much like exercise, such plant products possess the potential to affect the methylome, with several classes of these compounds shown to be able to reverse methylation, which could prove favorable in restoring normal gene expression (Fang, Chen, & Yang, 2007; Santos, Tewari, & Benite‐Ribeiro, 2014; Yang, Fang, Lambert, Yan, & Huang, 2008). In a study employing the administration of a tea saponin extract to mice fed a HFD, it was seen that treatment was able to enhance leptin action through increased POMC expression, which was attributed in part to mitigating hypothalamic inflammation, with the effect on DNA methylation not investigated (Yu et al., 2013). Also, synergistic effects of combining exercise and plant phytochemicals to intensify their individual metabolic effects in an animal model of established obesity and IR have also been reported (Lambert et al., 2018). Further evaluation of the combined potential of exercise and phytochemicals to modulate methylation patterns in diet‐induced models of obesity could be valuable.

Even with a multiplex of diet and exercise programs to treat obesity available, the potential of alternate and complementary medicinal therapies are increasingly being explored (Esteghamati, Mazaheri, Vahidi Rad, & Noshad, 2015). Obesity has been treated using acupuncture from ancient to modern times, with several reports showing its therapeutic efficacy on simple obesity (Belivani et al., 2013; Kokosar et al., 2018; Leng et al., 2018; Sui et al., 2012). In a study, Sprague Dawley rats were fed HFD and then treated with electroacupuncture (EA), followed by the examination of hypothalamic expression of obesity‐related factors. DIO increased tuberous sclerosis complex 1 (*TSC1*) promoter methylation which increased mammalian target of rapamycin expression leading to increased appetite, food intake, and weight gain (Leng et al., 2018; Zhang, Li, He, & Hu, 2015). This was due to increased expression of AgRP and NPY while POMC was downregulated. EA treatment promoted weight loss, with this being accompanied by the demethylation of the *TSC1,* leading to the inhibition of mammalian target of rapamycin complex 1. The *TSC1* gene regulates mammalian target of rapamycin signaling partly through perception of upstream stimuli such as intracellular and extracellular growth factors, environmental changes, energies, and nutrients; however, further studies evaluating the importance of *TSC1* methylation status in obesity are required (Leng et al., 2018).

## 9 FUTURE CONSIDERATIONS

Epigenetic mechanisms have increasingly been implicated in obesity pathology, and attempts at modification of these processes have rightfully been investigated as therapeutic strategies. An important feature of DNA methylation is that it is reversible (Ramchandani, Bhattacharya, Cervoni, & Szyf, 1999; Yang et al., 2008), with this characteristic making it an attractive therapeutic target for prevention and treatment of obesity and related illnesses. Indeed, some success has been reported in this regard, with studies showing that the manipulation of the epigenetic machinery, such as DNA methyltransferase‐1 (DNMT1) and DNA methyltransferase‐3a (DNMT3a), could be a useful strategy in treating obesity (Bruggeman, Garretson, Wu, Shi, & Xue, 2018; Kohno et al., 2014). The inhibition of DNMT1 using aurintricarboxylic acid was shown to result in reduced activity within the pancreatic β cells of T2DM mice, contributing to lowered blood glucose and improved insulin signaling, with this finding holding therapeutic promise for the treatment of diabetes (Chen, Liao, Tsai, & Tsai, 2016).

Further understanding of epigenetic mechanisms contributing to obesity‐related disease pathophysiology could be useful in assessing risk‐stratification and pave the way for the application of personalized therapies. Additional research aimed at exploring the possibility of reprogramming of maternal diet‐induced arcuate nucleus epigenetic phenotypes through postweaning interventions is still required. Although the health benefit of breastfeeding for both mother and child has long been known (Kramer & Kakuma, 2012), more awareness and encouragement are still required to draw attention to the importance of breastfeeding. Even though, according to World Health Organization, all infants should be exclusively breastfed from birth up to 6 months of age, most countries are still not doing enough to support mothers despite the potential health benefits as well as positive economic implications (Prell & Koletzko, 2016; Walters, Phan, & Mathisen, 2019). Breastfeeding is crucial to establish optimal or reference intake values for leptin during lactation, thereby designing personalized patterns of nutrition achieved through postnatal programming of a healthy phenotype in early childhood that will remain into adulthood (Palou, Pico, & Palou, 2018).

Furthermore, phytochemicals are known to affect epigenetics could thus represent suitable candidates in postweaning experimentation, with curcumin, genistein, epigallocatechin gallate, resveratrol, and equol able to inhibit DNMT activity (Ayissi, Ebrahimi, & Schluesenner, 2014), with such endeavors remaining largely unexplored. Additionally, gender differences observed in DNA methylation patterns, in the response to DIO as well as the action of plant compounds, have been observed (Ratnu, Emami, & Bredy, 2017; Shiina et al., 2019; Zore, Palafox, & Reue, 2018), highlighting the importance of considering gender for future research endeavors.

## 10 CONCLUSION

A high‐energy obesogenic environment can elicit epigenetic modifications that alter promoter DNA methylation patterns, thereby modulating gene expression. Diet‐induced DNA methylation changes occurring within ARC genes, particularly *POMC* and *INS‐R*, which prevents an appropriate response to leptin and insulin, thereby affecting energy homeostasis, subsequently leading to the development of obesity and MeS. Overnutrition in early stages of life via maternal diet can predispose offspring to the development of obesity throughout their lives. Through the implementation of healthy lifestyle practices, attempts to reverse aberrant DNA methylation of crucial arcuate nucleus genes could be useful in the treatment of obesity.

## CONFLICT OF INTEREST

The authors declare there is no conflict of interests.

## ETHICAL APPROVAL

This manuscript submission is in accordance with the stipulated publication ethical guidelines. This review article does not require ethical clearance since no animal and human testing was performed.
